# Rerefinement of the crystal structure of α-ThBr_4_

**DOI:** 10.1107/S2414314623008908

**Published:** 2023-10-17

**Authors:** Tim Graubner, Florian Kraus

**Affiliations:** a AG Fluorchemie, Fachbereich Chemie, Philipps-Universität Marburg, Hans-Meerwein-Strasse 4, 35032 Marburg, Germany; Vienna University of Technology, Austria

**Keywords:** crystal structure, thorium, thorium bromide, actinide bromide

## Abstract

Single crystals of α-ThBr_4_, thorium(IV) tetra­bromide, were obtained as a side product from the reaction of CuBr with β-ThBr_4_ at 753 K. In the crystal structure, the Th atom (site symmetry  ..) is surrounded by eight Br atoms in the form of a tetra­gonal-disphenoidal coordination polyhedron. The connectivity of these polyhedra is ^3^_∞_[ThBr_4/2_Br_4/2_]. In comparison with the previous crystal structure refinement [Mason *et al.* (1974 ▸). *J. Less-Common Met.* **35**, 331–338], the current rerefinement resulted in much higher preciscion of the lattice parameters and the atomic coordinates.

## Abstract 2

The rerefinement of the crystal structure of α-ThBr_4_ (α-ThCl_4_ structure type) results in higher precision of the lattice parameters and the atomic coordinates.

## Graphical abstract

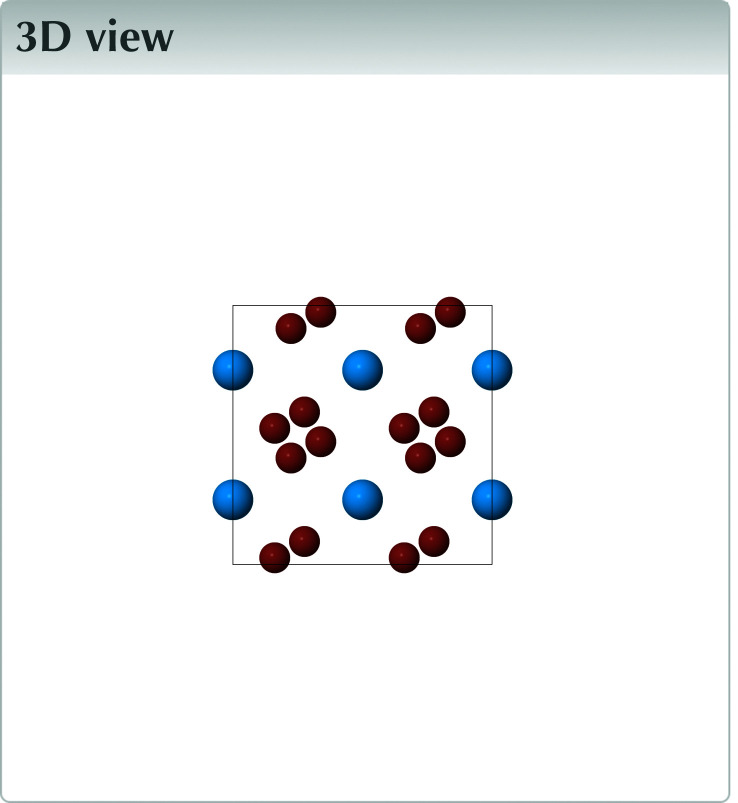

## Structure description

A crystal of ThBr_4_ in its α-modification was isolated as a side product from the reaction of β-ThBr_4_ with CuBr at 753 K.

The crystal structure of α-ThBr_4_ has been described only once, from a single-crystal X-ray diffraction study at room temperature (Mason *et al.*, 1974 ▸), where the authors refer to this modification also as the low-temperature polymorph. They reported the transition temperature at 699 ± 5 K and the crystal structure of α-ThBr_4_ was assigned to the α-ThCl_4_ structure type in the space group *I*4_1_/*a* (No. 88, *tI*20). A comparison of the structural parameters of the original crystal structure refinement and of the current rerefinement is given in Table 1 ▸.

**Figure 1:**
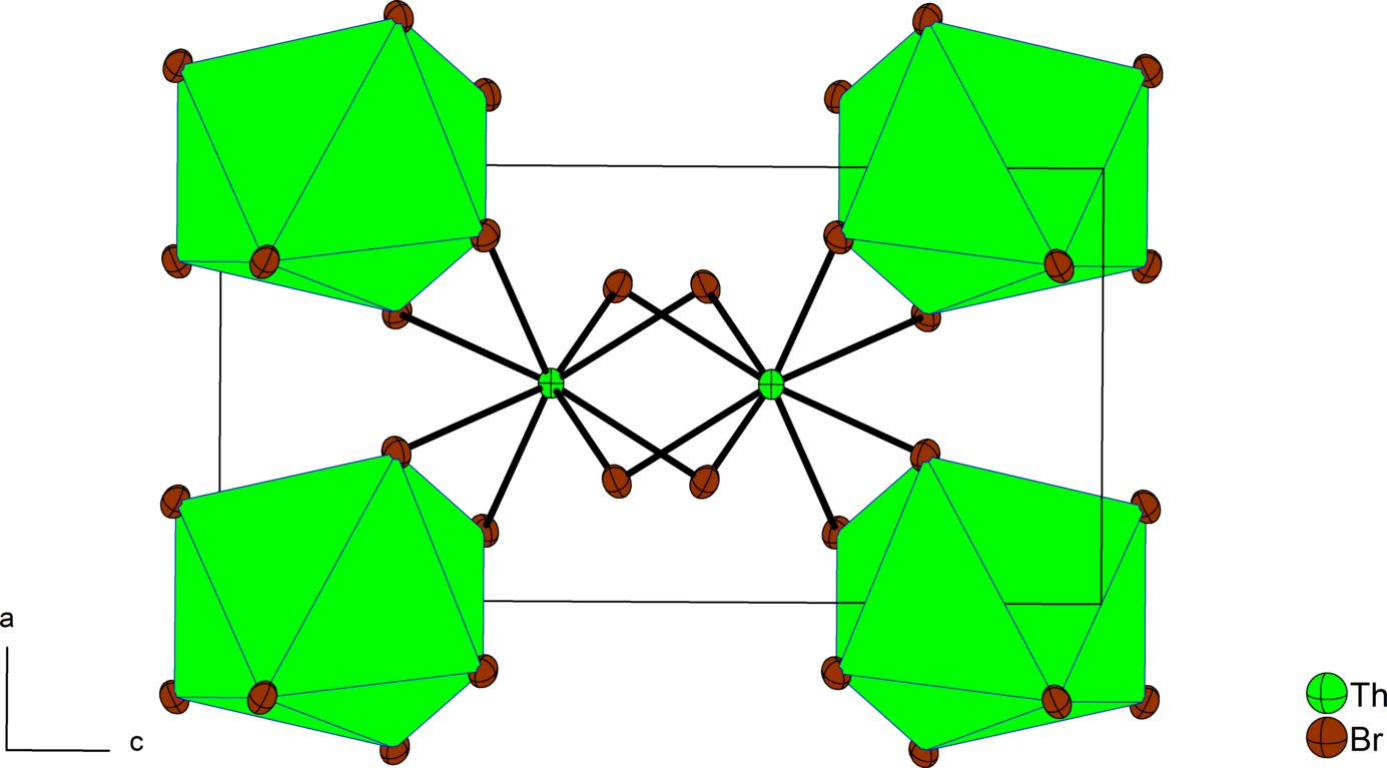
Crystal structure of α-ThBr_4_ in a projection along [010]. Displacement ellipsoids are drawn at the 90% probability level.

Fig. 1 ▸ shows the crystal structure based on the current X-ray diffraction data. There is one Th atom (multiplicity 4, Wyckoff letter *a*, site symmetry 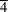 ..) and one Br atom (16*f*, site symmetry 1) in the asymmetric unit. The Th atom is surrounded by eight Br atoms to form a tetra­gonal-disphenoidal coordination polyhedron. The Th—Br bond lengths of 4 × 2.9100 (4) Å and 4 × 3.0107 (4) Å are in good agreement with previously reported values of 2.909 and 3.020 Å (no s.u. values or temperature given; Mason *et al.*, 1974 ▸), but different compared to those in β-ThBr_4_ (space group *I*4_1_/*amd*), with values of 2.85 and 3.12 Å (no s.u. values or temperature given; Brown *et al.*, 1973 ▸). Each Br atom bridges two Th atoms, which results in edge-sharing polyhedra to form the crystal structure. The connection motif of α-ThBr_4_ is similar to that in β-ThBr_4_. Although the two polymorphs differ considerably with respect to the two pairs of Th—Br distances, the connectivities in both structures can be described with the Niggli formula ^3^_∞_[ThBr_4/2_Br_4/2_]. The closest Th⋯Th distance of 4.77179 (12) Å in α-ThBr_4_ is shorter compared to β-ThBr_4_, with a value of 4.8774 Å (Brown *et al.*, 1973 ▸). In the crystal structure of α-ThBr_4_, each Th atom is surrounded by eight other Th atoms in the shape of an irreg­ular polyhedron, with Th⋯Th distances of 4 × 4.77179 (12) Å and 4 × 6.70680 (19) Å.

## Synthesis and crystallization

All work was carried under an argon atmosphere (5.0, Praxair) using a fine-vacuum line and a glove-box (MBraun). Silica ampoules were flame-dried under dynamic fine vacuum (10^−3^ mbar; 1 bar = 10^5^ Pa) at least three times before use. Aluminium bromide (Alfa Aesar, 98%) was sublimed *in vacuo* before use; β-ThBr_4_ was prepared according to a literature protocol (Deubner *et al.*, 2017 ▸).

A silica glass ampoule was loaded with β-ThBr_4_ (149 mg, 0.27 mmol) and CuBr (78 mg, 54 mmol, 2.01 equiv.), and sealed under vacuum. The ampoule was heated in a furnace to 753 K at a rate of 1 K min^−1^ and kept at this temperature for 480 h for the reaction to take place. Afterwards, it was cooled to 330 K at a rate of 50 K d^−1^. Several colourless crystals of α-ThBr_4_ were obtained.

## Refinement

Crystal data, data collection and structure refinement details are summarized in Table 2 ▸.

## Supplementary Material

Crystal structure: contains datablock(s) I, global. DOI: 10.1107/S2414314623008908/wm4200sup1.cif

Structure factors: contains datablock(s) I. DOI: 10.1107/S2414314623008908/wm4200Isup2.hkl

CCDC reference: 2300477

Additional supporting information:  crystallographic information; 3D view; checkCIF report

## Figures and Tables

**Table 1:** Comparison of structural parameters of α-ThBr_4_ resulting from the current and previous crystal structure refinements

	This work	Mason *et al.* (1974 ▸)
*a* (Å)	6.7068 (2)	6.737 (1)
*c* (Å)	13.5792 (6)	13.601 (3)
*x*, *y*, *z* Th	0, 1/4, 1/8	0, 1/4, 1/8
*x*, *y*, *z* Br	0.33880 (6), 0.47423 (6), 0.20021 (3)	0.3378 (6), 0.4727 (7), 0.1998 (3)

**Table 2:** Experimental details

Crystal data	
Chemical formula	ThBr_4_
*M*_r_	551.68
Crystal system, space group	Tetragonal, *I*4_1_/*a*
Temperature (K)	100
*a*, *c* (Å)	6.7068 (2), 13.5792 (6)
*V* (Å^3^)	610.81 (5)
*Z*	4
Radiation type	Mo *K*α
μ (mm^−1^)	50.43
Crystal size (mm)	0.15 × 0.15 × 0.14

Data collection	
Diffractometer	Bruker D8 QUEST
Absorption correction	Numerical (*SADABS*; Krause *et al.*, 2015 ▸)
*T*_min_, *T*_max_	0.016, 0.078
No. of measured, independent and observed [*I* > 2σ(*I*)] reflections	9305, 463, 463
*R*_int_	0.049
(sin θ/λ)_max_ (Å^−1^)	0.715

Refinement	
*R*[*F* ^2^ > 2σ(*F* ^2^)], *wR*(*F* ^2^), *S*	0.021, 0.053, 1.37
No. of reflections	463
No. of parameters	13
Δρ_max_, Δρ_min_ (e Å^−3^)	1.16, −1.72

Computer programs: *APEX3* and *SAINT* (Bruker, 2019 ▸), *SHELXT* (Sheldrick, 2015*a* ▸), *SHELXL* (Sheldrick, 2015*b* ▸), *DIAMOND* (Brandenburg, 2022 ▸) and *publCIF* (Westrip, 2010 ▸).

## full crystallographic data

### Crystal data

**Table:**

ThBr_4_	Melting point: 200 K
*M**_r_* = 551.68	Mo *K*α radiation, λ = 0.71073 Å
Tetragonal, *I*4_1_/*a*	Cell parameters from 9713 reflections
*a* = 6.7068 (2) Å	θ = 3.0–30.6°
*c* = 13.5792 (6) Å	µ = 50.43 mm^−^^1^
*V* = 610.81 (5) Å^3^	*T* = 100 K
*Z* = 4	Block, colorless
*F*(000) = 920	0.15 × 0.14 × 0.14 mm
*D*_x_ = 5.999 Mg m^−^^3^	

### Data collection

**Table:**

Bruker D8 QUEST diffractometer	463 independent reflections
Radiation source: Incoatec Microfocus	463 reflections with *I* > 2σ(*I*)
Multi layered optics monochromator	*R*_int_ = 0.049
Detector resolution: 10.42 pixels mm^-1^	θ_max_ = 30.5°, θ_min_ = 5.2°
φ and ω scans	*h* = −9→9
Absorption correction: numerical (SADABS; Krause *et al.*, 2015)	*k* = −9→9
*T*_min_ = 0.016, *T*_max_ = 0.078	*l* = −19→19
9305 measured reflections	

### Refinement

**Table:**

Refinement on *F*^2^	Primary atom site location: dual
Least-squares matrix: full	Secondary atom site location: difference Fourier map
*R*[*F*^2^ > 2σ(*F*^2^)] = 0.021	*w* = 1/[σ^2^(*F*_o_^2^) + (0.0219*P*)^2^ + 6.5321*P*] where *P* = (*F*_o_^2^ + 2*F*_c_^2^)/3
*wR*(*F*^2^) = 0.053	(Δ/σ)_max_ < 0.001
*S* = 1.37	Δρ_max_ = 1.16 e Å^−^^3^
463 reflections	Δρ_min_ = −1.72 e Å^−^^3^
13 parameters	Extinction correction: SHELXL (Sheldrick, 2015b), Fc^*^=kFc[1+0.001xFc^2^λ^3^/sin(2θ)]^-1/4^
0 restraints	Extinction coefficient: 0.0052 (4)

### Special details

**Table:**

Geometry. All esds (except the esd in the dihedral angle between two l.s. planes) are estimated using the full covariance matrix. The cell esds are taken into account individually in the estimation of esds in distances, angles and torsion angles; correlations between esds in cell parameters are only used when they are defined by crystal symmetry. An approximate (isotropic) treatment of cell esds is used for estimating esds involving l.s. planes.

### Fractional atomic coordinates and isotropic or equivalent isotropic displacement parameters (Å^2^)

**Table:**

	*x*	*y*	*z*	*U*_iso_*/*U*_eq_	
Th1	0.000000	0.250000	0.125000	0.00764 (15)	
Br1	0.33880 (6)	0.47423 (6)	0.20021 (3)	0.00953 (16)	

### Atomic displacement parameters (Å^2^)

**Table:**

	*U*^11^	*U*^22^	*U*^33^	*U*^12^	*U*^13^	*U*^23^
Th1	0.00844 (17)	0.00844 (17)	0.00604 (19)	0.000	0.000	0.000
Br1	0.0101 (2)	0.0104 (2)	0.0080 (2)	−0.00131 (13)	−0.00114 (13)	0.00179 (13)

### Geometric parameters (Å, º)

**Table:**

Th1—Br1	2.9100 (4)	Th1—Br1^iv^	3.0107 (4)
Th1—Br1^i^	2.9100 (4)	Th1—Br1^v^	3.0107 (4)
Th1—Br1^ii^	2.9100 (4)	Th1—Br1^vi^	3.0107 (4)
Th1—Br1^iii^	2.9100 (4)	Th1—Br1^vii^	3.0107 (4)
			
Br1—Th1—Br1^i^	138.907 (16)	Br1—Th1—Br1^vi^	72.606 (12)
Br1—Th1—Br1^ii^	97.075 (5)	Br1^i^—Th1—Br1^vi^	75.260 (8)
Br1^i^—Th1—Br1^ii^	97.075 (5)	Br1^ii^—Th1—Br1^vi^	72.605 (8)
Br1—Th1—Br1^iii^	97.076 (5)	Br1^iii^—Th1—Br1^vi^	148.466 (14)
Br1^i^—Th1—Br1^iii^	97.075 (5)	Br1^iv^—Th1—Br1^vi^	128.427 (10)
Br1^ii^—Th1—Br1^iii^	138.907 (16)	Br1^v^—Th1—Br1^vi^	75.934 (16)
Br1—Th1—Br1^iv^	148.466 (14)	Br1—Th1—Br1^vii^	72.605 (8)
Br1^i^—Th1—Br1^iv^	72.605 (8)	Br1^i^—Th1—Br1^vii^	148.466 (14)
Br1^ii^—Th1—Br1^iv^	72.606 (12)	Br1^ii^—Th1—Br1^vii^	75.260 (8)
Br1^iii^—Th1—Br1^iv^	75.260 (8)	Br1^iii^—Th1—Br1^vii^	72.606 (12)
Br1—Th1—Br1^v^	75.260 (8)	Br1^iv^—Th1—Br1^vii^	75.934 (16)
Br1^i^—Th1—Br1^v^	72.606 (12)	Br1^v^—Th1—Br1^vii^	128.427 (10)
Br1^ii^—Th1—Br1^v^	148.466 (14)	Br1^vi^—Th1—Br1^vii^	128.427 (10)
Br1^iii^—Th1—Br1^v^	72.605 (8)	Th1—Br1—Th1^vi^	107.394 (12)
Br1^iv^—Th1—Br1^v^	128.427 (10)		

Symmetry codes: (i) −*x*, −*y*+1/2, *z*; (ii) *y*−1/4, −*x*+1/4, −*z*+1/4; (iii) −*y*+1/4, *x*+1/4, −*z*+1/4; (iv) −*y*+1/4, *x*−1/4, *z*−1/4; (v) *x*−1/2, *y*, −*z*+1/2; (vi) −*x*+1/2, −*y*+1/2, −*z*+1/2; (vii) *y*−1/4, −*x*+3/4, *z*−1/4.
